# Evaluating an immunization carpool service for women in rural areas for facilitating routine childhood immunizations in Pakistan –a feasibility study on acceptability, demand, and implementation

**DOI:** 10.1016/j.jth.2024.101773

**Published:** 2024-05

**Authors:** Rozina Feroz Ali, Sundus Iftikhar, Mubarak Taighoon Shah, Vijay Kumar Dharma, Farrukh Raza Malik, Danya Arif Siddiqi, Subhash Chandir

**Affiliations:** a IRD Pakistan, 4th Floor Woodcraft Building, Korangi Creek, Karachi, 75190, Pakistan; b IRD Global, The Great Room, Level 10, One George Street, Singapore, 049145; c Establishment Division, Government of Pakistan, Pakistan

**Keywords:** Childhood immunization, Transport, Women-only, Access, Carpool service

## Abstract

**Introduction:**

Poor accessibility of immunization services coupled with limited options for transportation and socio-cultural norms that hinder women's mobility are among the key factors contributing to poor immunization coverage in rural areas. We assessed the feasibility and acceptability of establishing a free-of-cost, women-only carpool service for immunization in a rural setting in Pakistan and evaluated its preliminary impact on immunization coverage and timeliness among children.

**Methods:**

We conducted a feasibility study in four selected immunization facilities in Shikarpur District, Sindh. A local transport vehicle was hired and branded as an immunization carpool service. Women having un- or under-immunized children aged ≤2 years were invited to visit immunization facilities using carpool vehicles. Information on demographic indicators and service experience was collected. Child immunization details were extracted using the government's provincial electronic immunization registry to estimate immunization coverage and timeliness.

**Results:**

Between January and October 2020, six immunization carpool vehicles provided uninterrupted service and transported 2422 women-child pairs, completing 4691 immunization visits. Majority of women reported that the carpool service improved accessibility (99.6%) by offering group travel (82.9%) and reducing their dependency on family members (93.4%). Preliminary estimates reported an increase in immunization coverage and timeliness across antigens among participating children compared to non-participating children, with significant increase in proportion for BCG coverage (38.1%; p < 0.001, CI: 32.8%, 43.4%) and measles-2 timeliness (18%; p < 0.001, CI: 13.3%, 22.4%).

**Conclusion:**

A women-only immunization carpool service implemented within a rural setting is feasible and highly acceptable. Key factors contributing to the model's success include increased mobility and independence of women, cost-savings, and a culturally and contextually appropriate mechanism of transport embedded within the local setting. Increased accessibility to health services also contributed to improved immunization coverage and timeliness among children.

## Highlights

• Using local options, immunization carpool model can be introduced in rural areas.
• There is a high acceptance of free-of-cost, women-only transportation services.
• Women-only carpool transport options allow women to travel unchaperoned.
• Women-only immunization carpool improves zero-dose children coverage and overall immunization coverage and timeliness.

## 1 Introduction

Despite the proven benefits of routine childhood immunization in reducing child morbidity and mortality, 19.5 million children did not receive all basic vaccinations in 2018 globally. The COVID-19 pandemic pushed a further 5.9 million children below this threshold (World Health Organization, 2022). These aggregate numbers mask the substantial variation in the distribution of children missing vaccinations within and across countries. The majority of unvaccinated children are concentrated in remote, rural areas of low-middle income countries (LMICs), highlighting the inequities in access and uptake of immunization services in these settings (Palozzi et al., 2020)

Pakistan is among the top 10 countries with the largest burden of unvaccinated children and significant disparities in coverage rates within the country (World Health Organization, 2022). In 2018, only 63% of 0–23 month children in rural areas had received all essential vaccinations compared to 71% in urban areas (Pakistan Demographic and Health Survey 2017-18, 2019). Studies have mentioned various reasons for poor health coverage in rural areas, including a lack of essential opportunities such as education, stable livelihoods, health, and security (Hess, 2008). Coupled with this are the unique social values and cultural norms that undercut the gender roles within these areas and influence healthcare access and health-seeking behaviors. Constraints in access to healthcare facilities are a well-recognized barrier that leads to the underutilization of available healthcare services (Syed et al., 2013). Like other LMICs, unmet healthcare needs within rural areas of Pakistan are partly attributed to accessibility challenges. Poor road infrastructure, lack of transport facilities, restricted women's mobility, sparsely located immunization facilities, limited geographical reach, and socio-economic constraints are some of the prominent challenges in the rural areas of Pakistan (Jamal et al., 2020; Falagas et al., 2008; Hilber, 2010; Nirmal et al., 2012; Hjortsberg et al., 2002). Moreover, unpaved roads and a lack of affordable transport options hinder access to distantly located immunization facilities, contributing to poor immunization outcomes (Khowaja et al., 2015). Studies have reported a sharp decline in immunization coverage when the immunization facilities were located beyond 12 km of the child's residence (Jamal et al., 2020; Butt et al., 2020; Sajjad et al., 2017).

Accessibility constraints pertaining to mobility interplay with gender roles, cultural values, and socio-economic factors (Starkey et al., 2021). The existing socio-cultural norms in inherently patriarchal societies like Pakistan do not allow women to travel unchaperoned, particularly in rural settings (Adnan, n.d). The lack of commuting options further aggravates the concern since local transportation is predominantly utilized by males in rural communities and is limited to motorcycles, three-wheeled motorcycles (qingqis), and buses. Previous studies have demonstrated that women in rural areas prefer affordable, less crowded, safe, and timely transportation (Starkey et al., 2021). The absence of these facilities leads to mobility restrictions, preventing women from accessing facility-based healthcare services. Consequently, their dependence on partners and other family members for accompaniment and financial support is increased (Jamal et al., 2020; Sajjad et al., 2017).

Despite the necessity of culturally appropriate transport-related interventions to increase women's mobility for accessing health services in general and childhood immunization services in particular, it is among the more neglected and poorly addressed areas in literature (Butt et al., 2020). A few successful examples of non-emergency transportation interventions have been reported from developed countries that increased access and uptake of health services. For instance, a study in Pennsylvania showed a substantial influx of Medicaid patients at primary care units upon introducing a low-cost rideshare model (Chaiyachati et al., 2018). Moreover, providing free or subsidized rides through digital transport services such as Uber and Lyft increased access to COVID-19 vaccination centers in New Jersey, Illinois, California, and Virginia (Jercich, 2021; Bellon, 2021). These interventions were designed utilizing the already established transportation systems deployed in the countries and were usually part of a multi-component study. We did not find any studies on the impact of exclusive transport-related interventions from LMIC settings, including Pakistan. Our study contributes to the existing literature by filling a gap regarding the lack of information on women-only non-emergency transport services. This is crucial given the potential of the intervention to play a significant role in overcoming transportation barriers, respecting cultural norms, and empowering women, ultimately leading to increased and timely uptake of children's vaccinations. The purpose of the study was to design and implement a women-only carpool service for immunization in a rural setting and determine its acceptability and feedback from mothers and female caregivers of 0–23 months old children. A secondary objective was to generate preliminary evidence on the impact of carpool service on childhood immunization coverage and timeliness.

## 2 Methods

### 2.1 Study design and site

**Fig. 1:**
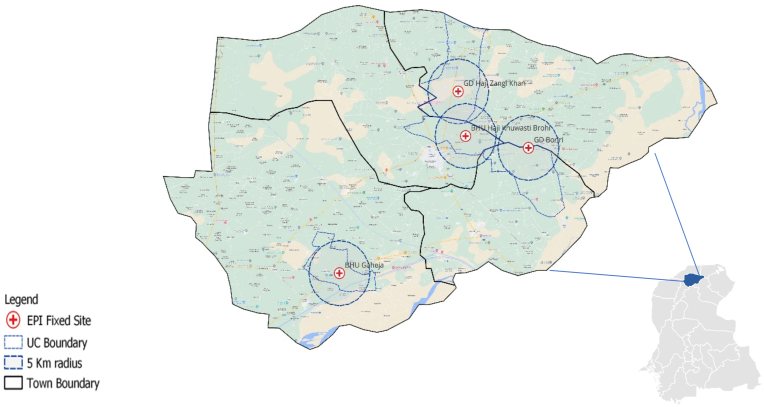
Map of District Shikarpur showing the four selected immunization facilities and their catchment areas.

The study was carried out in Shikarpur, Pakistan, a rural district spread over 2512 km^2^, with a population of 1,231,481 (Planning and Development Department, 2017). The district is divided into four sub-divisions, with 59 immunization facilities and 127 vaccinators (EPI, 2020). Each immunization facility is assigned a specific number of areas surrounding the facility called ‘catchment areas.’ We selected four immunization facilities, one from each sub-division, that catered to an estimated population of 40,000 people residing in 98 catchment areas of the four selected facilities (Fig. 1). The facilities were selected based on their low coverage rates and the unavailability of suitable public transportation options for commuting to the immunization facilities.

In 2020, the estimated full immunization coverage rate among children less than 12 months in Shikarpur was 30% (Government of Sindh Electronic Immunization Registry), less than half of the national coverage rate of 66%. Due to its rural geography, the district lacks a proper road network and transport system. The most prevalent commuting options in Shikarpur are personal or rented motorbikes and qingqis (three-wheeled vehicles that offer a ride-sharing commuting option).

### 2.2 Study population

The inclusion criteria for the study included children aged two years or below, as reported by their parents, who were due for any vaccine as indicated by the immunization status on their vaccination card or through maternal recall and were permanent residents of the catchment areas of the selected immunization facilities. Children ineligible to receive vaccines due to contradictions such as high-grade fever, infection, or history of vaccination-related adverse reactions reported by the child's caregivers were excluded.

Ethical approval for the study was obtained from the Institutional Review Board of Interactive Research and Development (IRB-IRD-201910017), which is registered with the US Department of Health and Human Services Office for Human Research Protections.

### 2.3 Study outcomes

Our study outcomes included the feasibility and acceptability of the immunization carpool model among women. For measuring feasibility, we examined the performance metrics of the carpool service, including its quality and regularity, the conduct of drivers, and the location of pickup and drop-off points. For measuring acceptability, we assessed the number of women who utilized the services to access immunization facilities for their child's vaccination. Further, their feedback regarding group traveling and vehicle branding was sought. Our secondary outcome was to assess the preliminary impact of the carpool service on immunization coverage and timeliness of children.

Coverage was analyzed for all antigens administered as part of the routine immunization schedule in Pakistan and the number of children who had received the antigen amongst all those who were due for the antigen. Timeliness was measured by determining the number of children who received the immunization on time per the WHO-recommended routine immunization schedule, followed by the Expanded Program on Immunization (EPI) Pakistan.

### 2.4 Study interventions

A free-of-cost, readily available, women-only immunization carpool service was introduced in catchment areas of selected immunization facilities. We conducted visits within the catchment areas to explore locally available transport options that could feasibly be adapted for the intervention. We selected Qingqis, a three-wheeler motorcycle with three forward-facing and three rear-facing passenger seats, as the immunization carpool vehicles since they were among the commonly used vehicles for commuting in the area. Furthermore, we conducted site mapping to record the distances of catchment sites from immunization facilities and identified key spots that were easily accessible by community members to serve as pickup and drop-off points for the carpool service. As a next step, we engaged the local transport providers to obtain six qingqis rented for the project duration. The qingqis were adapted for study use by pasting customized banners that provided information about the carpool services, the name of the nearest immunization facility, and facility timings. This aided in making the vehicles prominent, distinguishing them from other qingqis. Moreover, we produced a jingle in the local language that was played on the carpool vehicles during their operational hours to notify the community about the arrival of carpool vehicles in their area.

The community mobilization was done by conducting awareness sessions delivered by study staff to the catchment population at their houses or by assembling residents of 2–3 houses in one place. The key purpose of these sessions was to advocate for and inform about the immunization carpool services to community members, including males, to ensure their inclusion in health-related matters of their children and encourage female caregivers to utilize carpool services for attending immunization facilities. Moreover, banners were displayed at frequently visited places such as mosques, health facilities, and neighborhood shops, providing information about the newly introduced carpool services.

### 2.5 Study procedures and data collection

For the awareness sessions, trained study staff conducted household visits in the catchment areas of selected immunization facilities and conducted sessions with all the available community members aged 18 years or above. In particular, we approached those caregivers for the awareness sessions whose children were delayed on one or more of their vaccinations based on records extracted from the Government's Electronic Immunization Registry (EIR). For keeping a record of these sessions, logs were maintained with participants' details, including their identification, contact details, and the presence of ≤2 years children at home. Following the sessions, women in households with un- or under-immunized children aged ≤2 years were invited to utilize immunization carpool services to commute to the immunization facility for their child's vaccination. None of the caregivers refused to use the carpool service. The pick-up points and timings of the carpool service were also conveyed to the caregivers at this time.

After the awareness sessions, all willing women-child pairs were transported to the immunization facility through the carpool service. At the facility, study staff obtained verbal consent from women and captured information on the child's (and family's) demographic details, their access to vaccination facilities (cost and time), and commuting options. They also recorded the child's unique identification number assigned by the EPI, which was used as the unique study ID for the child. This unique ID allowed us to extract the child's immunization details from the Government's Electronic Immunization Registry (EIR) to assess the preliminary impact of carpool services on immunization uptake. After recording the demographic data, the children then proceeded for vaccination. Post-vaccination, the woman-child pairs were transported back to the designated drop-off points in the catchment sites. At the drop-off point, detailed feedback on the performance, perceived need, and acceptance of carpool services was collected by study staff from women, including their experience of the entire journey.

To capture the feasibility data, separate logs were maintained for each trip that recorded information on journey time, vehicle ID, driver, and the number of women-child pairs transported. In case of unavailability of designated carpool vehicles for any reason, the transport vendor provided a substitute transport vehicle with similar branding that helped ensure uninterrupted services.

During the COVID-19 lockdowns, special permissions were granted by public health officials to continue providing carpool services to communities during the lockdowns. Therefore, we amended the study protocols to comply with COVID-19 precautionary measures and continued facilitating immunization visits throughout the pandemic period.

### 2.6 Data analysis

Data from the paper forms were entered into the electronic database using Microsoft Access, version 16. Data from both questionnaires (one collected at the center and another at the drop-off point) was merged using the child's unique ID and date of visit. Less than 2% of observations for different variables were missing, which were included in the analysis without manipulation. Moreover, 2.7% of study children were found to be aged >2 years due to poor parental recall and were included in the study. For summary measures, we used frequencies (%) for categorical data and means and standard deviation (SD) for continuous data.

Immunization details were extracted from the government's EIR database, including vaccines administered, vaccination dates, and status for all children vaccinated at the four selected immunization centers during the intervention period. Using the child's EPI-ID from the study database, children were divided into participating groups (children who utilized the carpool services) and non-participating group (children who did not utilize the carpool service) groups. Antigen-wise immunization coverage and timeliness for both cohorts during the study period were calculated. The *t*-test was applied to estimate differences in coverage and timeliness between the two groups. Data analysis was done using STATA 17.0 software (StataCorp LLC StataCorp, TX).

## 3 Results

Between January 13 and October 31, 2020, six immunization carpool vehicles were deployed, facilitating 4691 immunization visits. These vehicles completed 2520 round trips for 2422 women-child pairs. On average, each vehicle completed 420 (Standard Deviation [SD]:58.2) round trips, served 398 (SD: 126) women-child pairs and facilitated 781 (SD: 171) immunization visits. We conducted 1100 awareness sessions with 3184 adult community members in catchment areas of the selected immunization facilities, 64% (2035/3184) of which were males.

### 3.1 Participants socio-demographic characteristics

Out of 2422 enrolled children in the study, 49.6% (1200/2422) were girls, and 49.7% (1204/2422) were under the age of 6 months. Around one-fifth of children (20.6%, 498/2422) received the BCG (Bacillus Calmette–Guérin) vaccine at enrollment, while a quarter (24.7%; 598/2422) attended the facility to get the first dose of the pentavalent vaccine (Table 1). Around one in every ten children enrolled in the study was zero-dose (8.6%, 208/2422), defined as a child who received the first dose of the pentavalent vaccine at or after one year of age. The mean age of mothers of enrolled children was 29 years (SD: 5.6), while the mean age of fathers was 33 years (SD: 6.6). In terms of the educational background of parents, 96.9% (2347/2422) of mothers and 75.2% (1821/2422) of fathers did not receive primary education. Most mothers (79.6%; 1929/2422) were homemakers, and fathers (87.8%; 2126/2422) were daily wage workers. The average monthly household income was USD 64.2 (SD: 26.3), while the average monthly travel expense of each household was USD 9.4 (SD: 6.4). The average monthly health-related travel expense was USD 4.8 (SD: 2.5) (Table 1). Three-fourths (78.3%, 1896/2422) of the children availing immunization carpool services were accompanied by their mothers (Table 2), while the remaining were accompanied by a woman relative (grandmother, aunt, or sister).

**Table 1:** Characteristics of children utilizing the carpool service (n = 2422).

Characteristics	n	%
**Child Age at Enrollment (months)**		
0–6	1204	49.7
7 - 12	561	23.2
13 - 18	410	16.9
19 - 24	181	7.5
>24	66	2.7
**Gender of child (Female)**	1200	49.6
**Father's Age (in years) (mean, SD)**	33.5 ± 6.6	
**Father's Education (years)**		
No education	1821	75.2
Primary	285	11.8
Secondary	168	6.9
Intermediate	118	4.9
Graduate & above	30	1.2
**Father's Occupation**		
Daily wages	2126	87.8
Self-employed	165	6.8
Others	131	5.4
**Mother's Age (in years) (mean, SD)**	29.4 ± 5.6	
**Mother's Education (years)**		
No education	2347	96.9
Primary	21	0.9
Secondary	28	1.2
Intermediate	18	0.7
Graduate & above	8	0.3
**Mother's Occupation**		
Homemaker	1929	79.6
Daily wages	339	14.0
Others	154	6.4
**Ethnicity**		
Sindhi	1609	66.4
Balochi	746	30.8
Punjabi	67	2.8
**Household Income (USD per month)**^a^**(mean, SD)**	64.2 ± 26.3	
**Travel Expense (USD per month) (mean, SD)**	9.4 ± 6.4	
**Health Related Travel Expenses (USD per month) (mean, SD)**	4.8 ± 2.5	
**Antigen Administered at Enrollment**^b^		
BCG	498	20.6
Penta-1	598	24.7
Penta-2	303	12.5
Penta-3	180	7.4
Measles-1	400	16.5
Measles-2	367	15.1
IPV (only)	30	1.2
TCV (only)	12	0.5
Not vaccinated	34	1.4
**Zero dose**^c^	208	8.6

a At an exchange rate of 160 (2020 rates).

b If child received multiple vaccines at enrollment, the earlier vaccine in the schedule is considered as enrollment vaccine.

c Child who has not received Pentavalent-1 at the age of 365 days.

**Table 2:** Information on access to immunization facilities reported by study participants (n = 2422).

Indicators	n	%
**Child accompanied to immunization center by**		
Mother	1896	78.3
Grandmother	222	9.2
Sister	171	7.1
Aunt	94	3.9
Other	39	1.6
**Mode of Transport used**		
Rented motorbike^a^	855	35.3
Walk	737	30.4
Three-wheeled motorcycles (Qingis)	671	27.7
Private vehicle	133	5.5
Other	26	1.1
**Commuting feasibility**		
Always travel alone	267	11.0
Always travel with someone	2149	88.7
Both	6	0.3
**Travel dependency**^b^		
Immediate family member	1493	69.3
Husband	458	21.3
Relative	145	6.7
Neighbor/friends	59	2.7
**Perceived carpool service need (yes)**	2413	99.6

a Used as means of public transportation.

b If not traveling alone (n = 2155).

### 3.2 Access to immunization services

Before the implementation of the intervention, women were traveling to the center using rented motorbikes (35.3%, 855/2422), walking (30.4%, 737/2422), or using qingqis (27.7%, 671/2422). Nine out of ten women (88.7%; 2155/2422) could not travel unchaperoned. Of them, 21.3% (458/2155) were commuting with their husbands, while 69.3% (1493/2155) with immediate family members other than husbands, 6.7% (145/2155) with other relatives, and 2.7% (59/2155) with neighbors. Most women (99.6%; 2413/2422) expressed the need for transportation to access immunization facilities (Table 2).

### 3.3 Feasibility and acceptability of immunization carpool service

The immunization carpool service operated uninterrupted throughout the study duration. Almost all participants endorsed that the carpool intervention was safe (92.7%, 2244/2422) and had accessible pickup and drop-off points (97.6%, 2364/2422) (Table 3). No complaints were reported by women on the conduct of drivers, and no unforeseen incidents with the vehicle or women were reported. Furthermore, no challenges were observed regarding any hindrances caused by male family members for women using the carpool service, highlighting its collective acceptability.

**Table 3:** Participants’ feedback on immunization carpool vehicles (n = 2422).

Indicators	n	%
**Advantages of immunization carpool services**		
Improves accessibility (yes)	2413	99.6
Save travel cost (yes)	1832	75.6
Save travel time (yes)	2283	94.3
Reduce dependency on family members (yes)	2263	93.4
Safe ride (yes)	2244	92.7
Women-only group travel (yes)	2009	82.9
**Travel cost saved by carpool services (USD)**^a^		
0	501	20.8
0.1–0.25	604	24.9
0.26–0.5	853	35.2
0.6–0.75	383	15.8
0.76–1	39	1.6
>1	42	1.7
**Time saved by carpool services (minutes)**^b^		
0	30	1.2
1–15	1536	63.5
16–30	638	26.3
31–45	71	2.9
46–60	101	4.2
>60	46	1.9
**Overall feedback on carpool services**		
Accessible pickup and drop-off spots (yes)	2364	97.6
Satisfied with service quality (yes)	2413	99.6
Appropriate branding (yes)	2403	99.2
Appropriate behavior of drivers (yes)	2364	97.6
Willingness to pay for services (yes)	31	1.3
**Reasons for utilizing carpooling services**		
Encouraged by study team (yes)	14	0.6
Importance of vaccination (yes)	2183	90.1
Free services (yes)	2341	96.7
Easy commuting option (yes)	2166	89.4

a At an exchange rate of 160 (2020 rates).

b Relative to any alternate means of commute.

Nearly all women (99.6%, 2413/2422) reported that the carpool service helped increase accessibility to EPI centers and reduced their dependency on family members (93.4%, 2263/2422). Moreover, most women liked the branding of vehicles (99.2%; 2403/2422) and were satisfied with the quality of carpool services (99.6%; 2413/2422) (Table 3).

More than half (77.5%, 1879/2422) of the study participants reported that immunization carpool service helped save travel costs up to USD 1, while one-fifth of the participants (20.8%, 501/2422) said that the services did not save any travel cost. Furthermore, 92.7% (2245/2422) of women stated that the carpool service saved their travel time up to 45 min, while 6.1% (147/2422) confirmed timesaving of more than 45 min per visit (Table 3).

Regarding the reasons for utilizing the immunization carpool service, most women reported free rides (96.7%, 2341/2422) and easy commuting (89.4%, 2166/2422) as reasons for availing the services. Nine out of ten women (90.1%; 2183/2422) also considered immunization being beneficial for their children as an underlying factor for service utilization (Table 3).

### 3.4 Preliminary evidence of improvement in immunization coverage and timeliness

The immunization coverage among participating and non-participating children enrolled at the same facilities was significantly different across antigens. The highest difference was observed for the proportion of BCG coverage of 38.1% (88.9% vs. 50.8%, *p* < 0.001, CI: 32.8%–43.4%), followed by the Penta-1 vaccine 30.9% (84.5% vs. 53.6%, *p* < 0.001, CI: 25.1%–36.6%) (Table 4).

**Table 4:** Comparison of antigen-wise coverage rates among participating and non-participating children at 4 study sites in district Shikarpur between January 13 to October 31, 2020.

Antigen	Coverage rate among participating children (n = 2422)			Coverage rate among non-participating children (n = 3177)			Difference	p-value	95% Confidence Interval
	Due (n)	Received (n)	%	Due (n)	Received (n)	%	%		%
BCG	920	818	88.9	400	203	50.8	38.1	<0.001	[32.8, 43.4]
OPV-0	553	552	99.8	202	136	67.3	32.5	<0.001	[26.0, 38.9]
Penta-1	859	726	84.5	345	185	53.6	30.9	<0.001	[25.1, 36.6]
OPV-1	858	728	84.8	342	183	53.5	31.3	<0.001	[25.5, 37.1]
PCV-1	876	737	84.1	346	181	52.3	31.8	<0.001	[26.0, 37.6]
Rota-1	849	713	84.0	327	181	55.4	28.6	<0.001	[22.7, 34.5]
Penta-2	1183	957	80.9	329	196	59.6	21.3	<0.001	[15.5, 27.0]
OPV-2	1182	952	80.5	330	196	59.4	21.1	<0.001	[15.3, 26.9]
PCV-2	1178	944	80.1	332	190	57.2	22.9	<0.001	[17.1, 28.6]
Rota-2	1169	926	79.2	324	189	58.3	20.9	<0.001	[15.0, 26.7]
Penta-3	1084	875	80.7	307	189	61.6	19.1	<0.001	[13.2, 25.0]
OPV-3	1082	873	80.7	305	187	61.3	19.4	<0.001	[13.4, 25.3]
PCV-3	1077	825	76.6	306	178	58.2	18.4	<0.001	[12.3, 24.5]
IPV	1074	758	70.6	356	172	48.3	22.3	<0.001	[16.3, 28.1]
Measles-1	1085	805	74.2	502	254	50.6	23.6	<0.001	[18.5, 28.6]
Measles-2	718	526	73.3	698	398	57.0	16.3	<0.001	[11.3, 21.1]

BCG: Bacillus Calmette-Guérin; OPV: Oral poliovirus vaccine; Penta, Pentavalent vaccine (Diphtheria, Tetanus, Pertussis, Hepatitis B, and *Haemophilus influenza* type b); PCV: Pneumococcal conjugate vaccine; Rota: Rotavirus vaccine.

Similarly, we also observed a difference in immunization timeliness between participating and non-participating children, with the highest difference in proportion of 18% between children vaccinated timely for measles-2 (25.5% vs. 7.5%, p < 0.001, CI: 13.3%–22.4%), followed by 10.8% difference in proportion of IPV vaccine (17.8% vs. 7.0%, p < 0.001, CI: 6.1%–15.5%) (Table 5). The absolute estimates showed that participating children received measles-2 dose 118 days earlier than non-participating children. For IPV, participating group was vaccinated 75 days earlier than non-participating group (Supplementary Table 1).

**Table 5:** Comparison of antigen-wise timeliness among participating and non-participating children at 4 study sites in district Shikarpur between January 13 to October 31, 2020.

Antigen	Timeliness among participating children (n = 2422)			Timeliness among non-participating children (n = 3177)			Difference	p-value	95% Confidence Interval
	Received (n)	Timely (n)	%	Received (n)	Timely (n)	%	%		
BCG	818	444	54.3	203	130	64.0	−9.7	0.012	[-17.1, −2.3]
OPV-0	552	441	79.9	136	120	88.2	−8.3	0.024	[-14.7, −1.9]
Penta-1	726	409	56.3	185	96	51.9	4.4	0.277	[-3.6, 12.4]
OPV-1	728	409	56.2	183	96	52.5	3.7	0.365	[-4.3, 11.8]
PCV-1	737	393	53.3	181	87	48.1	5.2	0.204	[-2.8, 13.3]
Rota-1	713	404	56.7	181	95	52.5	4.2	0.312	[-3.9, 12.3]
Penta-2	957	220	23.0	196	26	13.3	9.7	0.002	[4.2, 15.1]
OPV-2	952	219	23.0	196	26	13.3	9.7	0.002	[4.2, 15.1]
PCV-2	944	197	20.9	190	21	11.1	9.8	0.001	[4.6, 14.9]
Rota-2	926	214	23.1	189	27	14.3	8.8	0.007	[3.1, 14.5]
Penta-3	875	79	9.0	189	8	4.2	4.8	0.029	[1.3, 8.2]
OPV-3	873	79	9.0	187	8	4.3	4.7	0.031	[1.3, 8.2]
PCV-3	825	67	8.1	178	3	1.7	6.4	0.002	[3.7, 9.1]
IPV	758	135	17.8	172	12	7.0	10.8	<0.001	[6.1, 15.5]
Measles-1	805	298	37.0	254	73	28.7	8.3	0.015	[1.7, 14.7]
Measles-2	526	134	25.5	398	30	7.5	18	<0.001	[13.3, 22.4]

BCG: Bacillus Calmette-Guérin; OPV: Oral polio vaccine; Penta, Pentavalent vaccine (Diphtheria, Tetanus, Pertussis, Hepatitis B, and *Haemophilus influenza* type b); PCV: Pneumococcal conjugate vaccine; Rota: Rotavirus vaccine.

## 4 Discussion

This study demonstrates that there is high acceptance of culturally appropriate, free-of-cost, women-only group travel services among rural women in Pakistan, which improves their mobility and allows them to travel without male chaperones. The service can feasibly be established using local transport options, improving women's access to routine immunization services. Moreover, this study generated preliminary evidence of improved immunization coverage (between 16% and 38%) and timeliness (between 4% and 18%), for various vaccines administered as part of routine immunization schedule among participating and non-participating children who belonged to the same catchment sites. Timeliness for BCG and OPV-0 was lower among participating children indicating that these children were left behind and had not initiated their immunization process (were never-immunized) and would likely have remained unreached by the health system if they had not utilized the carpool service.

The study results corroborate existing findings in literature that show increased healthcare uptake when accessibility to health services is improved. Examples include non-emergency transport services designed to facilitate people with chronic diseases such as cancer, hypertension, and diabetes (Starbird et al., 2019), women seeking maternal care (Rajé, 2018), and medically insured individuals accessing clinics (Chaiyachati et al., 2018). A more recent example focused on immunization delivery included a ‘vaccine-ride’ initiative implemented in selected states of US to improve uptake and equity of COVID-19 vaccines in the country (Jercich, 2021; Bellon, 2021). It is noteworthy that, except for one, in all these studies, transport-related initiatives were part of a larger multi-component intervention. Therefore, our study uniquely evaluates an intervention focused entirely on a transportation service primarily designed for women only.

In addition to improving access to health services, it is pertinent to investigate the other complimentary pathways that contributed to the success of the immunization carpool service. One of the key factors was that the service was designed primarily for women. Existing literature highlights that women-only interventions not only improve women's health status but also that of their families, particularly their children (Midhet et al., 2010; Van Dammen et al., 2018; Saggurti et al., 2018). Women are usually considered the primary caregivers of children and are responsible for their child's health, including their immunizations (Rizkianti et al., 2020; Rosso et al., 2020). However, in most cases, they cannot seek health care for themselves and their children due to socio-economic constraints such as limited mobility, financial dependency, cultural norms, and poor knowledge (Sajjad et al., 2017; Abreha et al., 2021). Therefore, even when household attitudes and norms favor immunization, by virtue of men controlling critical resources, women nevertheless depend on men to enable access to these services. The carpool intervention essentially overcame these key challenges by enabling local women to accompany each other safely and access immunization services without depending on male family members. Additionally, the inclusion of males in awareness sessions (>50% of participants in these sessions were males) helped sensitize them about the safety of carpool intervention and resulted in better penetration of the intervention in the community. We postulate that involving male members in transport interventions targeted exclusively for women can also play a role in addressing the cultural norms that hinder female mobility. Some pertinent lessons from the study to increase take-up of the service include inclusion of male family members in the awareness process, increased community awareness for the intervention, maintenance of service quality and ensuring reliability of dropoff and pick-up times, as well as encouraging women users to share their positive experiences with their peer to reinforce the process of encouragement and take-up of the service.

Another distinguishing factor of the intervention was the adaptation of locally available and culturally acceptable means of transportation. Most of the existing transportation-related interventions in literature constitute ride-sharing models employing digital technology (Chaiyachati et al., 2018), provision of vouchers or coupons to avail public transport services (Percac-Lima et al., 2009; Kotwani et al., 2014), provision of free shuttle services (Percac-Lima et al., 2009), reimbursement of travel costs to attend clinics (Taylor et al., 2002; Kaplan et al., 2000) and provision of support for arranging transportation (Esperat et al., 2012; Krieger et al., 1999). In contrast to the above interventions that involved adapting complex processes or relying on digital tools or vouchers, the carpool intervention was inherently simple in nature and relied almost exclusively on existing resources easily available in the community study setting. The qingchis leveraged for this study were already being used as ambulances in case of emergencies in rural settings in Pakistan (Adnan, n.d). Furthermore, the target population in rural areas lacks critical resources such as data connectivity, smartphones, and technological proficiency to utilize more complex interventions. Therefore, we purposely designed the intervention to not rely on additional resources such as smartphones, internet connectivity, or complicated applications so that it could be embedded in rural areas effortlessly.

The carpool service introduced in this study provided free-of-cost services to women. In many countries, including Pakistan, routine vaccination programs are delivered through public health facilities that administer free vaccines to all children. Therefore, the main affordability barrier is traveling costs and the opportunity cost of taking time off from work to bring the child to the immunization center. This intervention essentially tackled both these barriers by making transportation free and not having to rely on male family members to bring children for immunization (over 87% of fathers in our study were daily wage earners vs. 14% of mothers). Over 75% of the respondents in the study stated that the intervention saved travel costs up to USD 1 per visit (adding up to USD 6 for a fully immunized child and USD 42 per family, given a fertility rate of 6.6 in the district) (Government of Sindh Electronic Immunization Registry). This is a substantial amount (even without accounting for the opportunity cost of wages foregone) considering high poverty rates in rural areas of the country.

From a public health policy perspective, this intervention provides evidence that it can improve the uptake and timeliness of immunization among children in remote rural areas. The data shows caregivers perceived the intervention as appropriate socio-culturally, resulting in its higher acceptability. Our study has generated the baseline evidence to steer future policy analysis. By applying Walt and Gilson's ‘Policy Triangle’ (Walt et al., 1994), our intervention has successfully identified the context (rural setting of LMICs), actors (caregiver and close family members, especially males), power dynamics (patriarchal outlook), content (women-only carpool service), and policy processes involved in the peculiar LMICs settings like Pakistan. Carpool intervention builds a strong case for the inclusion of free or subsidized women-only transportation facilities for improvement in accessing healthcare services, especially in remote, rural areas.

In addition to improving immunization coverage and timeliness, the intervention also has the potential to cover zero-dose children who otherwise could never get vaccinated due to various reasons such as inaccessibility or unwillingness to access immunization services (Mehmood et al., 2022). It is essential to note that these zero-dose children were not even covered by the immunization outreach teams that provide door-to-door vaccination services to children, showing caregivers' reluctance to vaccinate their children. The possible reasons for vaccine hesitancy could be fear of side effects such as post-vaccination fever and pain and vaccine-related myths and misconceptions (RF et al., 2022). Visiting immunization facilities allows interaction with fellow women attending clinics for their child's vaccination, which may help in reducing their fears pertaining to vaccinations. Moreover, occasionally, physicians are also available at primary health facilities who can provide appropriate information and counseling to mothers, eventually clarifying their myths and misconceptions related to vaccination. Hence, the socialization opportunity provided to women through carpool intervention would assist in addressing the leading causes of increasing zero-dose children. Therefore, it is suggested that immunization carpool services should be integrated into the government's zero-dose strategy and should be implemented in geographical clusters with a high proportion of zero-dose children.

Our study had a few limitations. First, the intervention was adapted during the pandemic because of which each vehicle transported only 1–2 women-child pairs per trip instead of the maximum capacity of 6. Additionally, fear of COVID-19 infection spread, lockdowns, and reduced mobility of people decreased the footfall at the immunization clinics during this time. Both the above points could have underestimated the overall benefits of the service. Secondly, the study was not designed to assess the impact of the intervention on immunization coverage and timeliness. However, we leveraged the EIR data of children vaccinated at the same centers but did not utilize the immunization carpool services as a surrogate control group. Hence, the estimates for immunization coverage and timeliness should only be considered preliminary evidence of the impact of the intervention. A key strength of the study is its replicability in terms of employing locally available resources, enabling the intervention to be easily adapted in rural and low-resource settings. As a way forward, we are considering the exploration of a rigorous pre-post design for conducting an impact evaluation study on this intervention. This approach will enable a comprehensive assessment of the carpool service's effectiveness, providing valuable insights for the future development and refinement of the intervention. Further work also needs to be done to include the perspectives of providers regarding the carpool service, addressing financial and other considerations.

## 5 Conclusion

The women's carpool model showed a high acceptance among rural women, allowing them to overcome cultural restrictions, limited mobility, and safety concerns. The women could safely travel in groups without financial and logistical dependency on males. The service can be established using local transport options, improving women's access to routine immunization and other health services. The study serves as baseline evidence for advocacy, policy analysis, and policy-making to improve healthcare delivery in rural areas of LMICs by providing free or subsidized commuting options to women. Moreover, the women's carpool has shown preliminary evidence of improving overall immunization coverage and timeliness and covering zero-dose children. Women carpool services could increase access to immunization services, vaccine equity, and uptake while reducing urban-rural disparities.

## Funding

This work was supported by the 10.13039/100000865Bill & Melinda Gates Foundation [grant number: OPP1212214].

## CRediT authorship contribution statement

**Rozina Feroz Ali:** Writing – original draft, Supervision, Resources, Project administration, Methodology. **Sundus Iftikhar:** Formal analysis, Data curation. **Mubarak Taighoon Shah:** Resources, Project administration, Methodology. **Vijay Kumar Dharma:** Resources, Project administration, Methodology. **Farrukh Raza Malik:** Writing – review & editing. **Danya Arif Siddiqi:** Writing – review & editing, Writing – original draft, Supervision, Methodology, Funding acquisition, Conceptualization. **Subhash Chandir:** Writing – review & editing, Writing – original draft, Supervision, Methodology, Funding acquisition, Conceptualization.

## Declaration of competing interest

All the authors of this article confirm that no personal, financial, or other related interests have influenced the findings presented in this paper.

## Data Availability

Data will be made available on request.
