# Supplementary material for: Evaluating an immunization carpool service for women in rural areas for facilitating routine childhood immunizations in Pakistan –a feasibility study on acceptability, demand, and implementation
Source: J Transp Health. 2024 May;36:101773. doi: 10.1016/j.jth.2024.101773 (PMC11256151; doi:10.1016/j.jth.2024.101773)
Supplement: Multimedia component 1 [file mmc1.docx]

**Supplementary Table**

| **Table 1:** Age at vaccination (in days) among children who utilized and did not utilize intervention at 4 study sites in district Shikarpur between January 13 to October 31, 2020 | | | | | | |
| --- | --- | --- | --- | --- | --- | --- |
|  | **Timeliness among children utilized intervention**  **(n=2,422)** | | **Timeliness among children**  **do not utilize intervention**  **(n=3,177)** | | **Mean**  **Difference** | **p-value** |
| **Antigen** | **Mean** | **(SD)** | **Mean** | **(SD)** |  |  |
|  |  |  |  |  |  |  |
| BCG | 65.1 | (98.7) | 53.5 | (89.3) | 11.6 | <0.001 |
| OPV-0 | 16.5 | (17.7) | 15.4 | (10.0) | 1.1 | 0.024 |
| Penta-1 | 117.1 | (96.0) | 126.5 | (113.6) | -9.4 | 0.003 |
| OPV-1 | 117.1 | (96.0) | 126.6 | (113.7) | -9.5 | 0.003 |
| PCV-1 | 118 | (96.4) | 126.6 | (113.2) | -8.6 | 0.008 |
| Rota-1 | 119.3 | (100.3) | 140.1 | (124.4) | -20.8 | <0.001 |
| Penta-2 | 180.9 | (117.8) | 224.7 | (163.8) | -43.8 | <0.001 |
| OPV-2 | 181.2 | (118.2) | 224.8 | (163.7) | -43.6 | <0.001 |
| PCV-2 | 183.9 | (118.9) | 225.7 | (164.5) | -41.8 | <0.001 |
| Rota-2 | 180.6 | (118.5) | 227.6 | (156.1) | -47 | <0.001 |
| Penta-3 | 246 | (128.6) | 317.5 | (175.5) | -71.5 | <0.001 |
| OPV-3 | 246.3 | (128.9) | 318.5 | (177.1) | -72.2 | <0.001 |
| PCV-3 | 250 | (130.5) | 318.6 | (176.6) | -68.6 | <0.001 |
| IPV | 226.2 | (117.4) | 300.8 | (168.2) | -74.6 | <0.001 |
| Measles-1 | 348.6 | (92.0) | 411.8 | (177.0) | -63.2 | <0.001 |
| Measles-2 | 543 | (117.4) | 661.1 | (173.5) | -118.1 | <0.001 |
| BCG: Bacillus Calmette-Guérin; OPV: Oral polio vaccine; Penta, Pentavalent vaccine (Diphtheria, Tetanus, Pertussis, Hepatitis B, and *Haemophilus influenza* type b); PCV: Pneumococcal conjugate vaccine; Rota: Rotavirus vaccine | | | | | | |
